# Slip-Flow and Heat Transfer of a Non-Newtonian Nanofluid in a Microtube

**DOI:** 10.1371/journal.pone.0037274

**Published:** 2012-05-15

**Authors:** Jun Niu, Ceji Fu, Wenchang Tan

**Affiliations:** State Key Laboratory for Turbulence and Complex Systems, College of Engineering, Peking University, Beijing, China; Massachusetts Institute of Technology, United States of America

## Abstract

The slip-flow and heat transfer of a non-Newtonian nanofluid in a microtube is theoretically studied. The power-law rheology is adopted to describe the non-Newtonian characteristics of the flow, in which the fluid consistency coefficient and the flow behavior index depend on the nanoparticle volume fraction. The velocity profile, volumetric flow rate and local Nusselt number are calculated for different values of nanoparticle volume fraction and slip length. The results show that the influence of nanoparticle volume fraction on the flow of the nanofluid depends on the pressure gradient, which is quite different from that of the Newtonian nanofluid. Increase of the nanoparticle volume fraction has the effect to impede the flow at a small pressure gradient, but it changes to facilitate the flow when the pressure gradient is large enough. This remarkable phenomenon is observed when the tube radius shrinks to micrometer scale. On the other hand, we find that increase of the slip length always results in larger flow rate of the nanofluid. Furthermore, the heat transfer rate of the nanofluid in the microtube can be enhanced due to the non-Newtonian rheology and slip boundary effects. The thermally fully developed heat transfer rate under constant wall temperature and constant heat flux boundary conditions is also compared.

## Introduction

### Nomenclature

*a* tube radius (m)


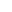
 shear stress (Pa)


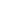
 pressure (Pa)


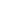
 axial velocity (m/s)


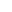
 axial location (m)


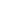
 radial location (m)

*n* flow behavior index of power-law fluid

*m* consistency coefficient of power-law fluid (N

s^n^/m^2^)


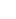
 slip constant (m)


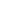
 shear rate (s^-1^)


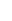
 critical shear rate at which the slip length diverges (s^-1^)


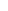
 density (kg/m^3^)


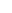
 specific heat of the nanofluid (J/kg

K)

*k* thermal conductivity (W/m

K)


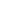
 density of nanoparticle (kg/m^3^)


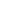
 specific heat of the nanoparticle (J/kg

K)


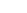
 specific heat of the base fluid (J/kg

K)


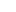
 temperature (K)


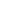
 constant entrance temperature (K)

*Nu* Nusselt number

*h* heat transfer coefficient (W/m^2^ 
K)


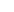
 slip radius

*r* dimensionless radial location

*T* dimensionless temperature

*z* dimensionless axial location

*Pe* Peclet number


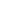
 average velocity (m/s)


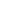
 eigenvalue


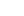
 eigenfunction


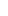
 nanoparticle volume fraction


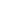
 volumetric flow rate (m^3^/s)

*Subscripts*

w wall

max maximum value

b bulk

fd fully developed

Nanofluid is the mixture of a base fluid (i.e., water, oil etc.) and solid nanoparticles with the diameter varying between 1 to 100 nm. Recently, the study of nanofluid is of growing interest for the observation of enhanced thermal conductivity [1], [2] which may throw light on the urgent cooling problems in engineering, for instance, the cooling of integrated circuits and micro-electromechanical systems. In order to use nanofluids for these small scale cooling techniques, the investigation on flow and heat transfer of nanofluids in microstructures like microtubes and microchannels is imperatively needed. As nanofluids are treated as homogeneous single-phase fluids (with the assumption that the nanoparticles are uniformly distributed in base fluids) in most related works, the most efficient way is to use the macroscopic results from the numerous existed studies on the flow and heat transfer of fluids in large scale structures. However, the macroscopic regulations may not be simply extended to apply for microscopic problems due to the appearance of some special phenomena with the shrinkage of characteristic length, e.g. the slip boundary. Experimental results [3], [4], [5] have shown that, in micro/nanoscale problems, the empirical non-slip boundary condition may break down depending on the fluid properties and interfacial roughness. Therefore, the slip effect should be taken into account which leads to the requirement of a slip boundary condition. The earliest slip boundary condition was proposed by Navier [6]. He showed a linear relationship between the slip velocity and the shear rate at the wall. But according to the results from molecular dynamics simulations, Thompson and Troian [7] discovered that the slip velocity is related to the slip length, the shear rate at the wall and a critical shear rate at which the slip length diverges.

Another important problem in the study of flow and heat transfer of nanofluid is the fluid rheology. One simplified way as has already been used in many works [8], [9] is to treat nanofluids as a Newtonian fluid with modified physical properties. However, recent experimental results have shown that the Newtonian model may not be accurate enough for describing the behavior of some particular nanofluids. Chang *et al.* [10] conducted an experiment for investigating the rheology of Cu-water nanofluid, in which they observed a shear-thinning fluid behavior. Similar phenomenon was also reported for the carbon nanotube-water nanofluid (CNT nanofluid) with the discovery that the increasing nanoparticle volume fraction makes the shear-thinning behavior more and more remarkable [11]. Pak and Cho [12] measured the viscosities of 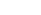-water and 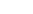-water nanofluids as functions of the shear rate. Their experimental results reveal that the appearance of the shear-thinning behavior depends on the species of the nanoparticle and the nanoparticle volume fraction. For the 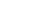-water nanofluid, the shear-thinning behavior emerges at a 3% nanoparticle volume fraction, but for the 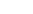-water nanofluid a higher 10% volume fraction is needed in order to display clear shear-thinning behavior. For describing the non-Newtonian shear-thinning rheology, the power-law model with the flow behavior index less than 1 is a good choice. Santra *et al.* [13] simulated the forced convection of Cu-water nanofluid in a channel with both Newtonian and non-Newtonian models. For the non-Newtonian case, the power-law rheology is applied in which the fluid consistency coefficient and the flow behavior index are interpolated and extrapolated from the experimental results with 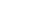-water nanofluid [14]. They discovered that the heat transfer rates calculated from the two models are different at high Reynolds numbers, which suggests that the non-Newtonian effect should be taken into account. Although many works have already been done on the flow and heat transfer of nanofluids, no one has considered the situation in a microtube with both non-Newtonian and boundary slip effects.

The objective of this work is to investigate the slip-flow and heat transfer of a non-Newtonian nanofluid in a microtube with constant wall temperature and constant heat flux boundary conditions.

For revealing the non-Newtonian effect on the flow and heat transfer characteristics, the rest of this paper is arranged as follows. We first derived the exact solution of the velocity profile and the series solution of the temperature distribution with constant wall temperature boundary. Then a validation of our techniques and calculating algorithm is conducted. Finally, the flow velocity, volumetric flow rate and local Nusselt number are calculated for different values of the nanoparticle volume fraction and slip length before a conclusion is drawn.

## Methods

### Model formulation

We consider the slip-flow and heat transfer of a nanofluid in a microtube with a radius *a*. The nanofluid is treated as a power-law non-Newtonian fluid with the classic Graetz assumptions of steady and incompressible flow, constant fluid properties, full developed velocity profile, negligible radial velocity and energy dissipation effects. The rheology can be expressed as
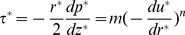
(1)where 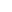 is the shear stress, 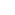 the pressure, 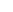 the axial velocity, 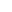 the axial location, 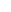 the radial location, *m*, *n* respectively the fluid consistency coefficient and the flow behavior index. The values of *m*, *n* are functions of the volume fraction of the nanoparticle. It should be noticed that in our work we consider the nanofluid as a single-phase fluid, so the slip we discuss is only between the nanofluid and the wall of the tube.

The slip boundary condition is introduced as in Thompson & Troian [7]

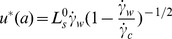
(2)where *a* is the radius of the microtube, 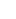 a constant slip length, 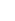 the shear rate at the wall of the tube, and 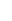 the critical shear rate at which the slip length diverges. Combining equations (1) and (2) followed by integration yields the velocity profile
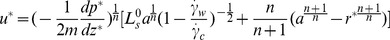
(3)For simplification, we made the same assumption as employed by Yang [15] that 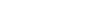, which may be reasonable in the slip-flow situation. The velocity profile is then
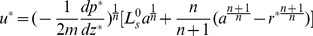
(4)with the maximum velocity at 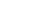 equaling
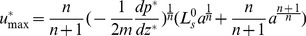
(5)


The energy equation is expressed as [16]

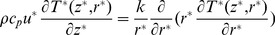
(6)where 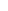,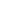,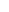 are respectively the density, specific heat and effective thermal conductivity of the nanofluid. From the definition of the nanoparticle volume fraction, the density of the nanofluid is
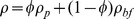
(7)where 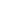, 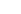 are the densities of the nanoparticle and the base fluid respectively. If we assume that the nanoparticles and the base fluid are in thermal equilibrium, the specific heat should be calculated as [17]

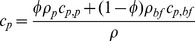
(8)in which 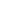 , 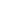 are respectively the specific heats of the nanoparticle and the base fluid. The following correlation [17] is used to calculate the effective thermal conductivity of the nanofluid, which is based on the experimental measurements [12], [18].
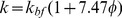
(9)where 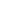 is the thermal conductivity of the base fluid.

Here we consider first the heat transfer of the non-Newtonian nanofluid with the constant wall temperature boundary. The case with constant heat flux boundary will be discussed later. The boundary condition reads
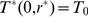
(10a)

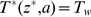
(10b)where 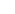 and 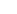 are the constant entrance temperature and constant wall temperature respectively. It should be noticed that we neglect the energy dissipation and the axial heat conduction in light of the classical Graetz assumptions. The temperature jump at the wall is also assumed to be negligible, same as in the works of Barron *et al.* [19] and Barkhordari & Etemad [20].

The important quantities for investigating the heat transfer characteristics of the nanofluid are the bulk temperature 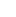 and the local Nusselt number *Nu* defined as
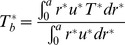
(11)and
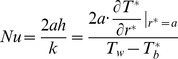
(12)where *h* is the heat transfer coefficient.

For dealing with the slip boundary condition, a slip radius 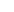 is derived from the non-dimensionalized velocity profile *u* [16]

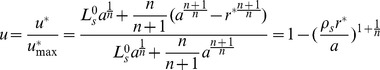
(13)where 
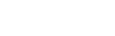
 (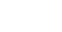). With the dimensionless variables 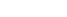, 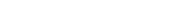 and 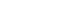, the energy equation can be non-dimensionalized as
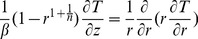
(14)Here, 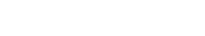. The Peclet number 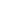 is defined as 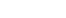, where 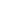 is the average velocity. The dimensionless boundary conditions are
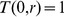
(15a)

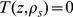
(15b)According to the radial symmetry,
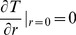
(16)The dimensionless bulk temperature and the local Nusselt number are respectively rewritten as
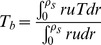
(17)and
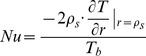
(18)


### Solution for temperature distribution

For solving equation (14), a separation-of-variables technique is used. We easily obtain a series solution of equation (14) as
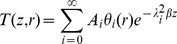
(19)where 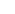 are constants, 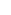 are the eigenvalues, and 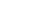 are the corresponding eigenfunctions satisfying the following equation
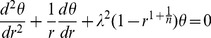
(20)with the boundary conditions coming from equation (15b) and equation (16)
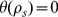
(21)and
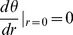
(22)Hence, the problem is reduced to determine the eigenvalues and the corresponding eigenfunctions. The Ritz method [21] is used for solving equations (20)–(22). While taking equation (20) as the Euler equation, the desired variational formulation can be expressed as
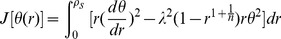
(23)


We assume the solution of equation (20) as a power series
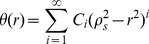
(24)where 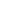 are constants. Equations (21) and (22) are automatically satisfied. This solution should make equation (23) reach the extreme value, which means by substituting equation (24) into equation (23), there exist the following equalities
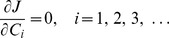
(25)


Equation (25) can be rearranged as
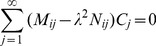
(26)where
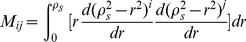
(27a)

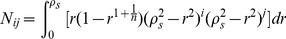
(27b)


To get a non-trivial solution series 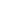, the coefficient determinant of equation (26) must be zero, viz.,
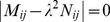
(28)equation (28) is a polynomial in the square of the eigenvalue from which 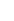 can be determined. For each solution of equation (28), let us say 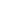, a series of 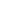 is obtained from equation (26), thus the corresponding eigenfunction can be determined
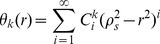
(29)By substituting equation (15a) into equation (19), one can easily obtain the following relation
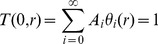
(30)It should be noticed that equation (20) is of the Sturm-Liouville type with the weight function being 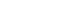, therefore, the following equality exists
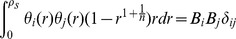
(31)where 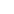, 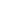 are normalization constants, and 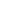 is the Kronecker delta. Then, by combining equations (30) and (31), the coefficients 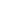 are gained [22]

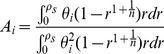
(32)With the above derivation, the velocity profile and a semi-analytical solution of the temperature distribution are obtained which will be used for discussing respectively the flow and heat transfer characteristics of the nanofluid with a slip boundary condition.

### Test case

**Figure 1:**
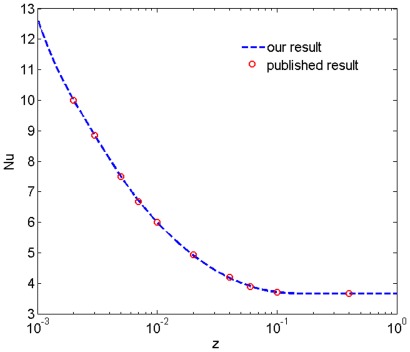
Validation of our algorithm. We compare our local Nusselt number of a non-slip Newtonian fluid in a tube with that in the previous work for testing the algorithm.

A numerical algorithm is employed to perform the above techniques for calculating the temperature distribution of the nanofluid in a microtube with a slip boundary condition. For validating that our techniques and algorithm are applicable and correct, we compare our results of the local Nusselt number with published results for a non-slip Newtonian fluid in a microtube [16] by setting the flow behavior index *n* equal to 1 and the slip length 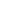 to be zero. In order to get the same dimensionless energy equation, we also assume the value of *Pe* to be 1. Figure 1 exhibits the calculated local Nusselt number for 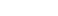 with the infinite series of the temperature solution in equation (19) being truncated at *n* = 10, the published results are also plotted for comparison. It can be seen that our calculated result is in perfect agreement with that in the published work. Therefore, our techniques and algorithm are applicable to the present problem.

## Results and Discussion

For investigating the characteristics of flow behavior and heat transfer of the nanofluid in a microtube, the flow velocity, volumetric flow rate, temperature distribution and the local Nusselt number are typical variables which should be considered. In our model, besides the pressure gradient, there exist another two parameters affecting the value of these four variables: one is the volume fraction of the nanoparticle 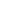 which is related with the fluid consistency coefficient *m* and the flow behavior index *n*; the other is the slip length 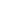 representing the slip effect. So in the following discussion, we first study the influence of these two parameters on the flow of the nanofluid by analyzing the variation of velocity profile and volumetric flow rate with 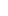 and 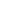 respectively; then, for revealing their effects on the heat transfer of the non-Newtonian nanofluid, the axial temperature distribution (a ten-term solution form which can provide enough accuracy for the current problem) and the local Nusselt number are calculated. The 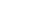-water nanofluid is used as an example in the following calculation with the values of *m*, *n* listed in Table 1 as functions of 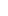 [13], [14].

**Table 1:** Values of the fluid consistency coefficient *m* and flow behavior index *n* for different 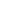 [13], [14].

nanoparticle volume fraction 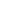 (%)	m (N sec^n^ m^−2^)	n
0.0	0.00100	1.000
1.0	0.00230	0.830
2.0	0.00347	0.730
3.0	0.00535	0.625
4.0	0.00750	0.540
5.0	0.01020	0.460

### Influences of nanoparticle volume fraction and slip length on the flow of the nanofluid

**Figure 2:**
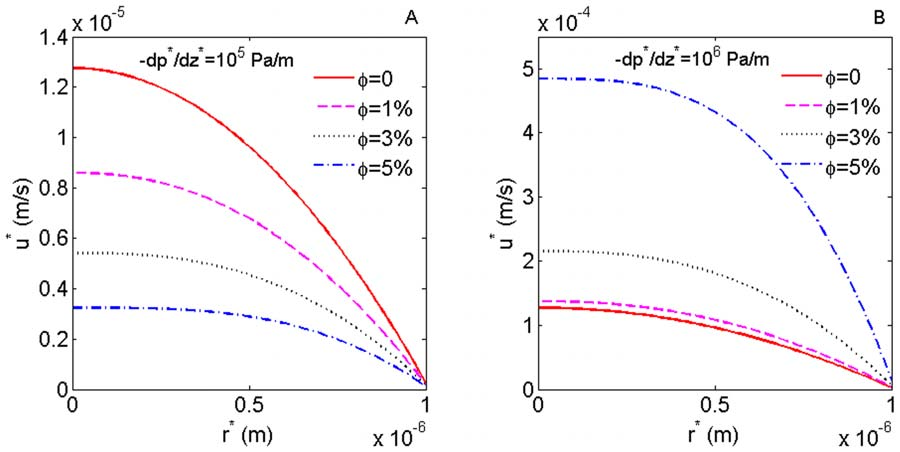
The velocity profiles for different nanoparticle volume fraction and pressure gradient. (**A**) The velocity profiles for 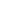
0, 1%, 3% and 5% with the pressure gradient equaling 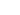 Pa/m. (**B**) the velocity profiles for 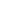
0, 1%, 3% and 5% with the pressure gradient equaling 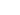 Pa/m.

It is shown in equation (4) that the flow velocity is proportional to the pressure gradient and the slip length. However, the dependence of the velocity on the nanoparticle volume fraction (which is related to *m*, *n* as shown in Table 1) is not easy to find at a glance. If the nanofluid is treated as a Newtonian fluid, the corresponding dynamic viscosity 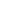 has been proven to increase with an increasing 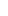 [23]. It can thus be deduced from the Poiseuille law that the increase of 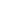 can decrease the velocity. However, due to the existence of the non-Newtonian effect, we discover that the influence of the value of 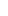 on the flow velocity depends on the value of pressure gradient. For illustrating this interesting phenomenon, we first fix the microtube radius as 1 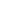
m and the slip length as 10 nm. Figure 2(a) shows half of the velocity profiles for 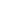
0, 1%, 3% and 5% with the pressure gradient equal to 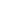 Pa/m. Clearly, the flow is slowed down with the increase of 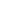. But for a larger pressure gradient 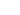 Pa/m, the flow velocity becomes larger with the increase of 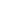, as illustrated in figure 2(b). For further analyzing this phenomenon, the variation of the volumetric flow rate will be shown as a function of the pressure gradient.

**Figure 3:**
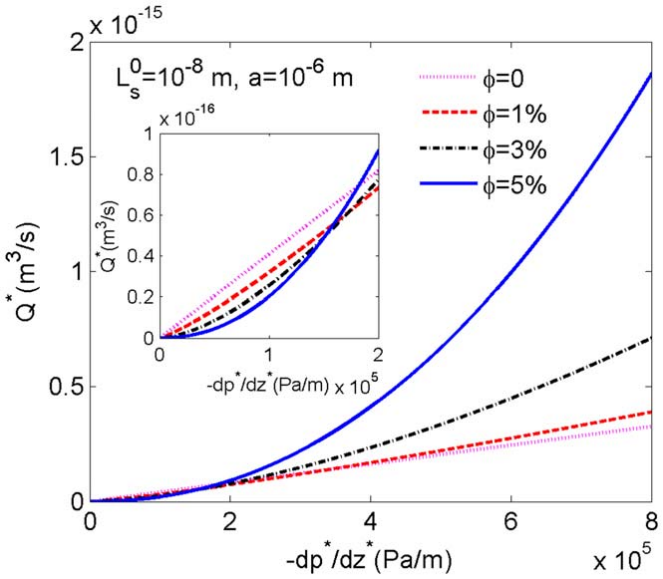
The volumetric flow rate of the nanofluid in a thin tube. We calculate the volumetric flow rates as a function of the pressure gradient for 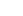
0, 1%, 3% and 5% with radius 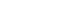 and slip length 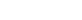.

Integrating equation (4) over the radial cross section of the microtube gives the volumetric flow rate as
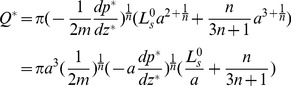
(33)We assume the radius of the microtube to be 1 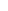
m and the slip length is fixed as 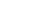 nm. The variation of 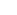 with the pressure gradient 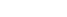 is plotted in figure 3 for 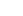
0, 1%, 3% and 5%, respectively. It should be noticed that the case with 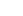
0 represents a pure water case, so the Newtonian rheology is applicable in which the volume flow rate is proportional to the pressure gradient. The inset in figure 3 shows the enlarged part of the figure up to 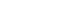 = 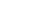 Pa/m. Clearly, for any fixed pressure gradient smaller than about 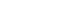 Pa/m, the flow rate decreases with the increase of 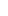. But as the pressure gradient increases, the curves for 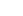
1%, 3%, 5% bend upward, while the curve for 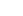
0 is a straight line due to the Newtonian rheology. Finally, the curve with a larger 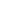 becomes higher when the pressure gradient is large enough, which is contrary to that in the small pressure gradient case, and the interval between two adjacent curves keeps increasing with the increase of the pressure gradient. This phenomenon can be interpreted from the expression of 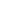. It is shown in Table 1 that with the increase of 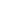, the value of the fluid consistency coefficient *m* increases while the value of the flow behavior index *n* decreases, these opposite varying directions make the variation of the value of 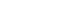 in equation (33) not large. So for any fixed pressure gradient, the relationship between 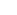 and 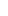 highly depends on 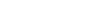 since 1/*n* is the index. When 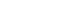 is much smaller than 1, the decreasing *n* resulting from the increase of 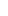 has the effect to decrease the flow rate which corresponds to the inset of figure 3. But as 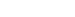 becomes much larger than 1, the decreasing *n* can increase the value of 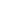, which means at a large pressure gradient adding more nanoparticles can facilitate the flow.

**Figure 4:**
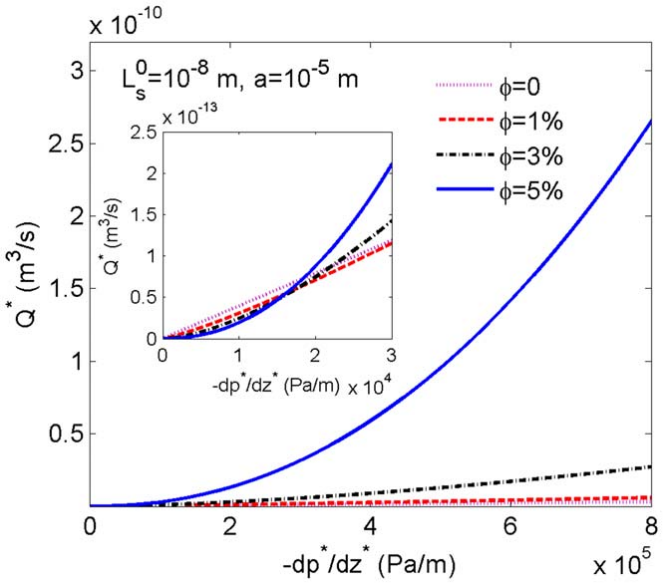
The volumetric flow rate of the nanofluid in a thicker tube. The volumetric flow rates as a function of the pressure gradient are calculated for 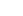
0, 1%, 3% and 5% with radius 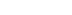 and slip length 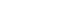.

We can deduce from the above discussion that the range of pressure gradient in which the increasing 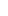 has the effect to resist the flow highly depends on the radial dimension of the microtube, which also reveals the existence of scale effect. When the radius *a* increases to 10 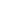
m, as shown in figure 4, the curves for 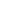
0, 1%, 3% and 5% exhibit similar phenomenon as that in figure 3, but the inset shows that the critical pressure gradient (below which 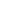 decreases with the increase of 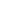) decreases to about 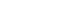 Pa/m. Finally, when *a* is as large as several centimeters, the critical pressure gradient becomes very small and the flow rate can be considered as increasing with the increase of nanoparticle volume fraction, contrary to the results based on the Newtonian model.

It is shown in equation (33) that 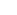 increases with the increase of 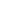 provided that the pressure gradient and volume fraction are fixed. Therefore, a larger value of the slip length, which represents a more remarkable slip effect, can promote the nanofluid flow. The ratio of the first term to the second term in equation (33) equals 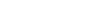, which denotes the relative flow rate of the slip-controlled flow to the pressure-gradient-driven flow. As 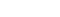 has the magnitude of O(1) in our problem, the slip-controlled flow must be taken into account if the value of slip length 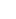 is comparable with the radius of the microtube. But when 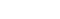, the slip-controlled flow becomes negligible and the non-slip boundary condition is accurate enough to describe the fluid flow.

### Influence of nanoparticle volume fraction on the heat transfer of the nanofluid

**Figure 5:**
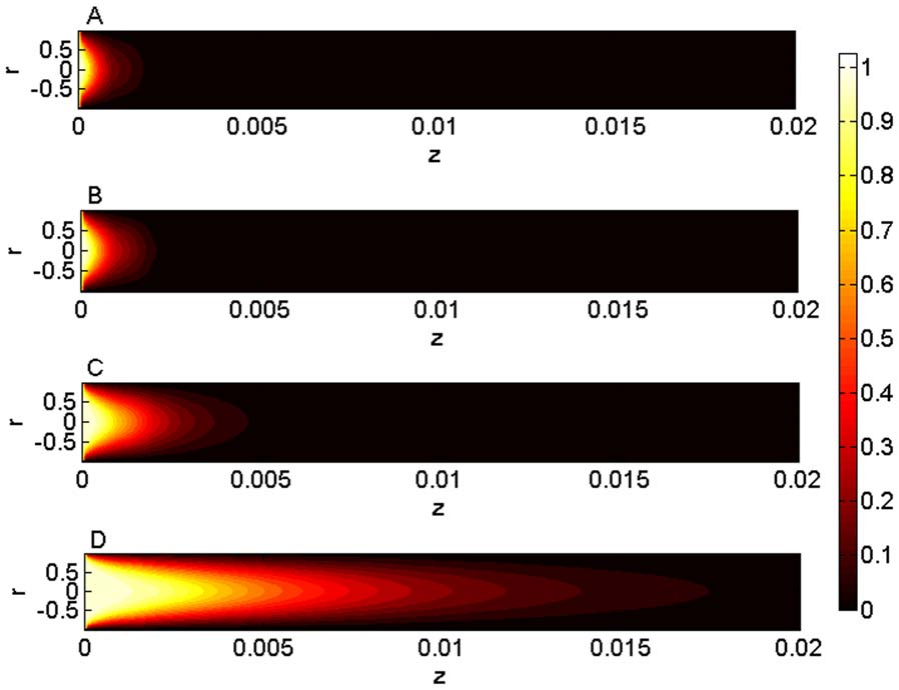
The temperature profiles of the nanofluid corresponding to different nanoparticle volume fractions. (**A**)–(**D**) Comparison of the axial temperature profiles for 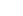
0, 1%, 3% and 5% with a fixed pressure gradient equal to 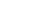 Pa/m and dimensionless slip length 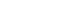, showing the effect of nanoparticle volume fraction on the temperature distribution.

The influence of the nanoparticle volume fraction on the heat transfer of the non-Newtonian nanofluid is investigated with fixed relative slip length 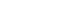. We first calculate the temperature distribution with a large pressure gradient 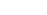 Pa/m. Figure 5 (a)–(d) show the axial temperature profiles for 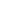
0, 1%, 3% and 5% respectively. Obviously, with the existence of non-Newtonian effect, the length of the thermal entrance region increases with the increase of 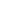 due to the larger volumetric flow rate. A larger volumetric flow rate also results in smaller thickness of the thermal boundary layer, which has the effect to increase the temperature gradient near the wall, so does for the heat transfer rate in the thermal entrance region.

**Figure 6:**
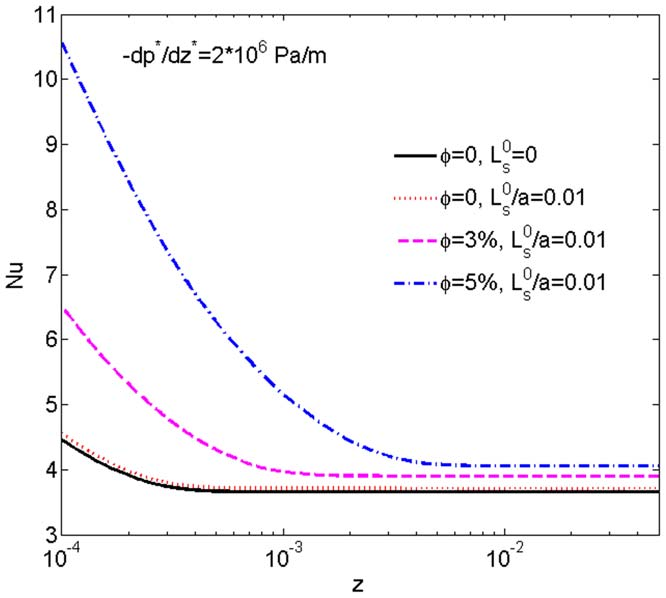
The heat transfer rates corresponding to different nanoparticle volume fractions. The heat transfer curves are calculated for 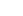
0, 3% and 5% with a fixed dimensionless slip length 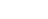
0.01 and pressure gradient 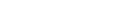 Pa/m, showing the effect of nanoparticle volume fraction on the heat transfer rate; the non-slip curve for 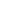
0 is also plotted for revealing the slip effect.

With the same fixed pressure gradient 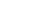 Pa/m and relative slip length 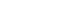, we also calculated the local Nusselt number *Nu* for 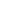
0, 3% and 5% as a function of *z* in figure 6. It can be seen that for any fixed *z*, the value of *Nu* increases with the increase of 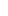. So adding nanoparticle can enhance the heat transfer rate. Moreover, according to our results with 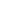
5% and 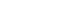, the Nusselt number in the fully developed region is 9.3% higher than that of a Newtonian nanofluid under the same conditions. This is resulted from the non-Newtonian effect, as for a Newtonian nanofluid in a small tube, with the classic Graetz assumptions, the heat transfer rate in the fully developed region is a function of the slip length only, independent of the nanoparticle volume fraction [16]. For comparison, the curve of local Nusselt number for a non-slip Newtonian fluid in a tube with a constant wall temperature is also plotted in figure 6, from which the Nusselt number in the fully-developed region is determined to be 3.65, in excellent agreement with published result [24]. Furthermore, this curve also reveals that the slip effect enhances the heat transfer rate which will be discussed in detail in the next subsection. For the case with a small pressure gradient (i.e., 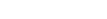 Pa/m), though not shown here, we found that the length of the thermal entrance region and temperature gradient near the wall decrease with the increasing 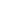, which is caused by the decreasing volumetric flow rate with the increase of 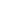, resulting in suppressed heat transfer rate near the entrance. However, the thermal entrance region is very short in the small pressure gradient case due to the very small fluid velocity; the local heat transfer rate in this region thus has limited effect on the averaged heat transfer rate. We have checked the local Nusselt number in the fully developed region, which is found to keep increasing with the increase of 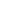 (the fully-developed local Nusselt number, on the other hand, is independent of the pressure gradient). Therefore, it is reasonable to conclude that adding nanoparticles can enhance the averaged heat transfer rate of the non-Newtonian nanofluid in a microtube.

### Influence of slip length on the heat transfer of the nanofluid

**Figure 7:**
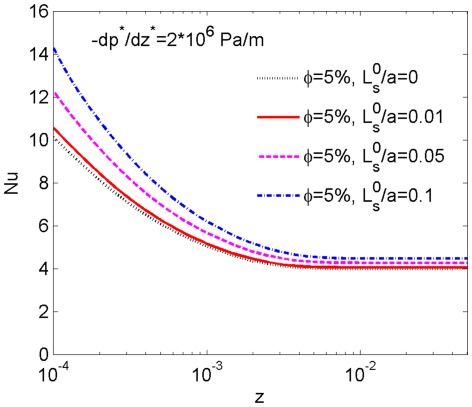
The heat transfer rates corresponding to different boundary slip lengths. Comparison of the heat transfer curves for 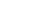
0, 0.01, 0.05 and 0.1 with a fixed nanoparticle volume fraction 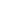
5% and pressure gradient 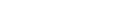 Pa/m, showing the effect of slip length on the heat transfer rate.

In order to investigate the influence of the slip length on the heat transfer of the non-Newtonian nanofluid, we first fix the nanoparticle volume fraction as 5% and the pressure gradient as 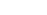 Pa/m, then the local Nusselt number *Nu* is calculated with different values of the relative silp length 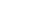. Figure 7 exhibits the variation of *Nu* as a function of z for 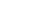
0, 0.01, 0.05 and 0.1, respectively. One can easily see from figure 7 that the local Nusselt number decreases with the increase of z until it falls to the fully developed value, and the value of slip length has little effect on the length of thermal entrance region. For any fixed z, the value of *Nu* increases with the increase of the dimensionless slip length. This can be interpreted from the variation of the nanofluid velocity at the wall. It is shown in equation (4) that the velocity at the wall increases with the increase of the slip length if the pressure gradient and the nanoparticle volume fraction are assumed to be constants, and an increasing boundary slip velocity can facilitate the heat transport between the nanofluid and the wall. So the increase of the value of slip length can promote heat transfer of the nanofluid.

### Comparison of the fully developed heat transfer rates of the nanofluid with constant wall temperature and constant heat flux boundary conditions

**Figure 8:**
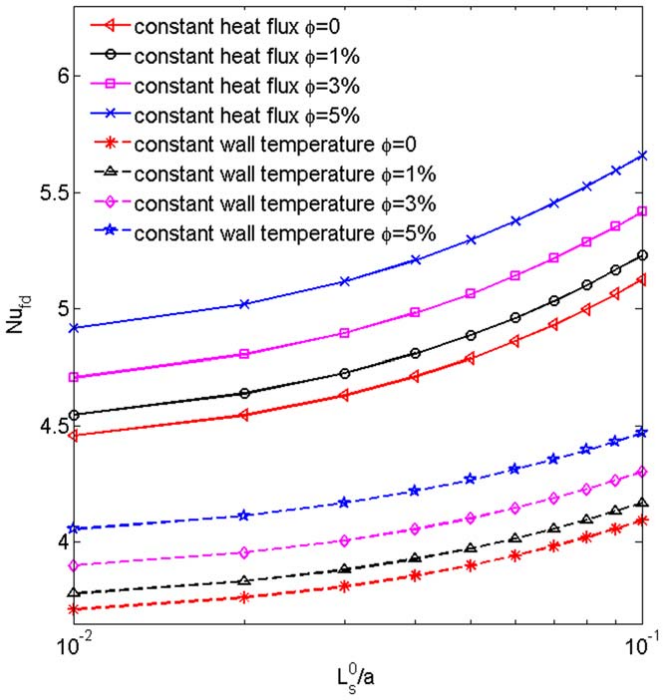
The thermally fully developed heat transfer rates for different boundary conditions. The thermally fully developed Nusselt numbers for 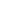
0, 1%, 3% and 5% as a function of the slip length with both constant wall temperature and constant heat flux boundary conditions are compared.

For studying the effect of thermal boundary condition on the heat transfer rate of the nanofluid in a microtube, we plot in Fig. 8 the thermally fully developed Nusselt number 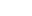 for the cases with constant wall temperature and constant heat flux boundary conditions as a function of the slip length. The derivation of 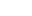 under constant heat flux boundary condition is similar to that in the book by Zhang [25] and will not be repeated. One can see under both boundary conditions the heat transfer rate is enhanced with the increase of the nanoparticle volume fraction and slip length. With fixed values of 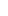 and 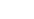, 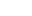 for the case with constant heat flux is always larger than that for the case with constant wall temperature. Moreover, with an identical 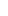 the distance between the two curves corresponding to the two types of boundary conditions increases with the increase of slip length.

In summary, the slip flow and heat transfer of a non-Newtonian nanofluid in a microtube is studied in light of the classic Graetz assumptions. We first calculate the velocity and volumetric flow rate of the nanofluid as a function of the pressure gradient with different nanoparticle volume fractions. Then the axial temperature profiles of the nanofluid under constant wall temperature boundary are obtained with the power series solution of the temperature distribution. Finally, the local Nusselt number representing the heat transfer rate of the nanofluid was determined. The results revealed the influences of nanoparticle volume fraction and slip length on the flow and heat transfer of the nanofluid with the existence of non-Newtonian effect. First, with a fixed slip length, the increase of the nanoparticle volume fraction can decrease the flow rate at a small pressure gradient; but as the pressure gradient becomes large enough, the flow rate increases with the increase of the nanoparticle volume fraction. This remarkable phenomenon is observed when the radius of the tube shrinks to micrometer scale. Second, for any fixed pressure gradient and slip length, the averaged heat transfer rate increases with the increase of the nanoparticle volume fraction. Third, the increase of slip length can enhance the heat transfer rate of the nanofluids. Finally, effect of thermal boundary condition on the thermally fully developed heat transfer of the nanofluid is studied; results show the Nusselt number for the case with constant heat flux boundary is always higher than that with constant wall temperature boundary.
